# Quantitative Metrics for Performance Monitoring of Software Code Analysis Accredited Testing Laboratories

**DOI:** 10.3390/s21113660

**Published:** 2021-05-24

**Authors:** Wladmir Araujo Chapetta, Jailton Santos das Neves, Raphael Carlos Santos Machado

**Affiliations:** 1 Division of Metrology in Information Technologies and Telecommunications, Brazilian National Institute of Metrology, Quality, and Technology (Inmetro), Av. Nossa Sra. Das Graças 50, Duque de Caxias, Rio de Janeiro 25.250-020, Brazil; wachapetta@inmetro.gov.br; 2 Institute of Computing, Praia Vermelha Campus, Fluminense Federal University (UFF), Boa Viagem—Niterói, Rio de Janeiro 24.210-346, Brazil; jailton_neves@id.uff.br

**Keywords:** proficiency testing, interlaboratory comparisons, accredited laboratories, software product evaluation

## Abstract

Modern sensors deployed in most Industry 4.0 applications are intelligent, meaning that they present sophisticated behavior, usually due to embedded software, and network connectivity capabilities. For that reason, the task of calibrating an intelligent sensor currently involves more than measuring physical quantities. As the behavior of modern sensors depends on embedded software, comprehensive assessments of such sensors necessarily demands the analysis of their embedded software. On the other hand, interlaboratory comparisons are comparative analyses of a body of labs involved in such assessments. While interlaboratory comparison is a well-established practice in fields related to physical, chemical and biological sciences, it is a recent challenge for software assessment. Establishing quantitative metrics to compare the performance of software analysis and testing accredited labs is no trivial task. Software is intangible and its requirements accommodate some ambiguity, inconsistency or information loss. Besides, software testing and analysis are highly human-dependent activities. In the present work, we investigate whether performing interlaboratory comparisons for software assessment by using quantitative performance measurement is feasible. The proposal was to evaluate the competence in software code analysis activities of each lab by using two quantitative metrics (code coverage and mutation score). Our results demonstrate the feasibility of establishing quantitative comparisons among software analysis and testing accredited laboratories. One of these rounds was registered as formal proficiency testing in the database—the first registered proficiency testing focused on code analysis.

## 1. Introduction

Conformity assessment is a fundamental activity for industry and society [1]. Based on activities such as technical standardization, metrology, testing, calibration, certification and accreditation—which are jointly known as the national quality infrastructure—it is possible to provide assurance that products, processes, people and management systems meet standard technical requirements. In countries where a solid national quality infrastructure is established, the activities related to conformity assessment are carried out by bodies that meet requirements described in the ISO/IEC 17000 family of standards. One of these standards applies, in particular, to calibration and testing laboratories: to have their calibrations and tests nationally and internationally recognized, these laboratories must meet the requirements of the current ISO/IEC 17025 and be accredited by an official national accreditation body [2,3].

One of the key requirements that provide assurance on the calibration and testing performed by accredited laboratories is that of *performance monitoring*. In the current ISO/IEC 17025:2017 [2], performance monitoring is defined in item 7.7.2, according to which an accredited laboratory shall “monitor its performance by comparison with results of other laboratories”. The ISO/IEC 17025:2017 standard also states that performance monitoring shall include participation in the so-called *interlaboratory comparison*, which is a formal procedure to compare the results of two or more laboratories. One approach for interlaboratory comparison is proficiency testing. For an interlaboratory comparison to be recognized as a proficiency testing, a series of requirements defined in the current ISO/IEC 17043 [4] must be met, including the prior publication of all criteria for approval or disapproval of laboratories. The rationale behind an interlaboratory or proficiency testing is that, if a laboratory shows inconsistent results when compared to other labs, this can evidence problems with its testing and calibration methods.

Proficiency testing has been for a long time [5,6,7] a well-used tool for the evaluation of accredited laboratories operating in the areas of physics, chemistry and biology. Currently, Information and Communication Technology (ICT) and Software Engineering (SE) have evident importance for most industries. As a consequence, practitioners and researchers have developed a set of technical standards and documents for the most diverse subjects in these domains [8,9,10]. Furthermore, many countries have developed several ICT or software related conformity assessment programs, aiming at certificating people, management systems, processes and products according to technical standards and regulations. However, proficiency testing programs for laboratories operating in the field are not yet available. There are challenges in developing software proficiency testing, for instance: (1) software products are not tangible; (2) requirements frequently accommodate some level of ambiguity, inconsistency or information loss; and (3) no metrics exist to uniquely characterize and identify them. Thus, even though software verification and validation activities involve several reasonable objectives, tools and methods, there is still a great influence of human variables (also known as soft factors) [11,12,13] in the execution of tests, including the team members’ prior experience.

In the present work, we evaluate the use of two quantitative metrics in order to make the objective comparison between the participating laboratories feasible. We propose metrics based on concepts from SE: code coverage [14] and software mutation [15]. The proposed approach was evaluated by executing two interlaboratory comparison rounds with the participation of software analysis and testing accredited laboratories and demonstrated the feasibility of establishing quantitative comparison among those labs. One of the executed rounds of interlaboratory comparisons followed all the requirements of ISO/IEC 17025:2017(E) and was registered in the international EPTIS proficiency resting database.

The remainder of the paper is organized as follows. In Section 2, we provide the basic definition and concepts for this work. In Section 4, we propose a metric based on code coverage for interlaboratory comparison and describe the implementation of a proficiency testing round based on code coverage. In Section 5, we propose a metric based on software mutation for interlaboratory comparison and describe the implementation of an interlaboratory comparison round based on software mutation. Section 6 contains our final considerations.

## 2. Background: Definitions and Concepts

### 2.1. Quality Infrastructure

*Quality infrastructure* is a framework comprised of metrology, standardization, verification, tests and quality activities and has a fundamental role for national and international trade because it is based on two types of standards: (1) measurement standards, embodying and communicating units of measure; and (2) standards to describe how to use these units, how to perform measurements and how to carry out other technical and managerial tasks. While national standards assure that measurements are comparable and reliable throughout a country, international standards ensure common measurements across borders and facilitate international trade. In general, nations having such an infrastructure use accreditation and conformity assessment activities to determine whether services and products meet established quality requirements [1,16,17,18].

*Accreditation* is the process by which a country’s official accreditation body formally recognizes the competence of a conformity assessment body. In the case of testing laboratories involved in conformity assessment activities, the accreditation is a declaration that the lab fulfills the requirements of ISO/IEC 17025 or national equivalent standard, in addition to the recognition of the technical capacity to perform a set of tests in the scope of the activities of the laboratory. Besides, the accreditation process, in conjunction with international mutual recognition agreements, may give international validity to the tests carried out by an accredited laboratory among their signatories. Essentially, the role of accredited laboratories is carrying out tests to demonstrate that a given product meets a set of requirements normally associated with a regulation or technical standard such as ISO standards [16,19].

Maintaining a solid national network of accredited laboratories is a challenge. Such laboratories must combine technical excellence in each of their accredited scopes with a solid management system that guarantees the quality—and the recognition—of the results of their tests and calibrations. This combination of technical excellence and quality assurance is essential for adequately supporting the industry and the mutual recognition of tests, calibrations and conformity assessment activities among the countries sharing equivalent levels of technical barriers in certain product certification programs.

### 2.2. Interlaboratory Comparison

Accredited laboratories must demonstrate their technical excellence to ensure the validity of the results of their tests and calibrations. Thus, for periodically comparing the laboratories’ measurements, a well-consolidated practice in empirical science is a fundamental activity to ensure the aforementioned validity and the confidence within the national quality infrastructure, so much so that it is emphasized by the ISO/IEC 17025:2017 (E) standard in its article 7.7.2. The importance of interlaboratory comparison led ISO Conformity Assessment Committee (CASCO) to develop a specific standard for conducting so-called proficiency testing, ISO/IEC 17043:2010 [4], describing all the requirements for an interlaboratory comparison to have international recognition—although ISO/IEC 17025:2017 (E) admits that a laboratory monitors its performance through interlaboratory comparisons distinct from proficiency tests formally defined in ISO/IEC 17043.

While accreditation is accepted as a proof of a laboratory’s competence, proficiency testing and other interlaboratory comparisons demonstrate the current performance and quality of test and calibration results of laboratories [20]. Traditionally, researchers and nations have developed and employed well-studied and grounded methods to undertake them, for instance, in biology [21], chemistry [22] and physics [23].

### 2.3. Software Testing and Information Technology Standards

The demand for regulation—and subsequent conformity assessment programs—for software-controlled devices and components has grown significantly in the last years [24,25,26,27,28].

In general, the methods most commonly used in the software conformity assessment are similar to those used by the manufacturer itself in software verification and validation activities [29]. Thus, different software testing techniques, such as inspections, functional testing, integration testing, system testing, etc. [30], can be used to determine whether a particular software product meets the established requirements. Software testing can be seen as a set of activities and actions to plan and provide inputs, focusing on certain abstraction levels and characteristics of the product and verify whether it responds as expected. However, both inputs and expected responses need to be defined a priori and compared to the product’s observed behaviors in reality. When the observed behaviors do not match the expected responses, product defects and faults have been found. However, testing a software product is admittedly complex [31].

ISO/IEC/IEEE 29119 [32] recommends a set of practices for software development organizations, which synthesized specifications from other previously published standards, such as IEEE 829, IEEE 1008, BS 7925-1 and BS 7925-2. The new standard met market demands despite criticisms and contrary movements, because several managers were demanding the standardization of testing processes that would guarantee the best functioning of this sector, but, in relation to the standard, the main concerns of the testing community were as follows [32]:

- lack of consensus between committee and testing community;
- need for extensive and difficult to implement documentation provided for in the standard;
- plastering or reversing the testing process by adopting the standard; and
- impact on other areas of the cycle that were not tested.

According to Computer Security Division [26], ISO/IEC 24759 extracts the requirements from ISO/IEC 19790, and, to ensure that the requirements are met, it associates supplier information and laboratory procedures. The CMVP (Cryptographic Module Validation Program) manages the variations allowed in ISO/IEC 19790 and ISO/IEC 24759 through SP 800-140x documents, provide additional evidence and tests to meet the evidence of CMVP cryptographic module requirements and provide adjustments recommended by ISO/IEC to the existing standard in the next revision. SP 800-140A through SP 800-140F provides additional requirements for supplier evidence, security policy, approved encryption and key management, authentication and non-invasive physical security requirements.

**Figure 1:**
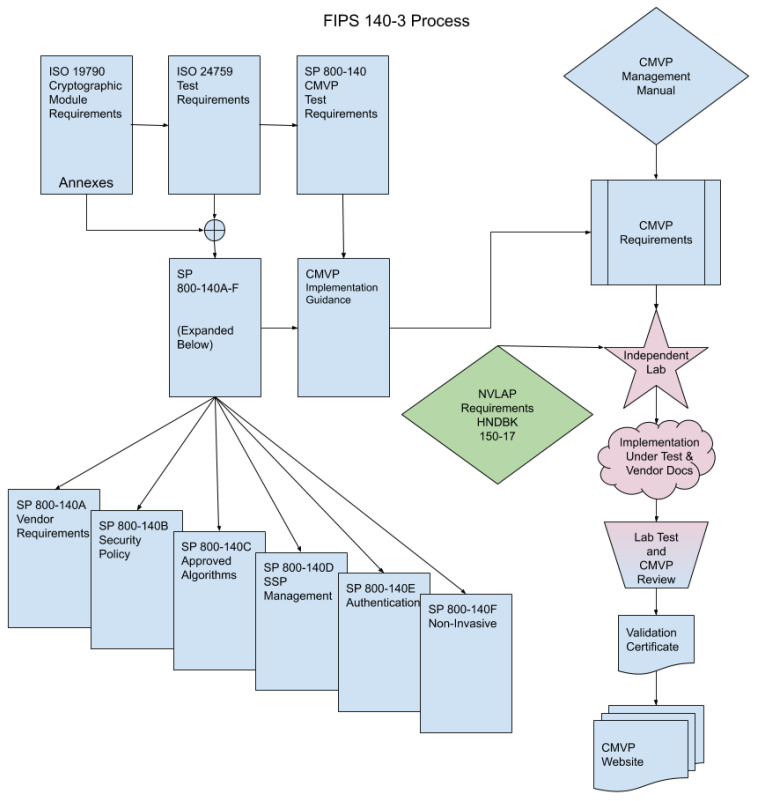
FIPS 140-3 process flow [26].

Another important document is the FIPS 140-3 CMVP management manual, which deals with programmatic procedures and process requirements. It also has the NVLAP Handbook 150-17 that identifies the NVLAP requirements specific to the CMVP, such as requirements in quality systems, personnel, environmental conditions, test and calibration methods, equipment, test quality assurance and control of reported results. Independent test laboratories with appropriate NVLAP accreditation manage and carry out the testing process and create a package with the results of the evaluation, which the CMVP reviews and then coordinates the resulting comments with the laboratory. After a satisfactory agreement, a validation is issued and added to the database hosted on the CMVP website [26]. Figure 1 illustrates the flow of requirements for the FIPS 140-3 process.

Furthermore, software conformity assessment introduces a series of new challenges arising from the degree of subjectivity associated with the interpretation of the requirements of a software product, as well as on the different understandings regarding how software can be analyzed or tested [19,30]. As a result, several requirements of ISO/IEC 17025 need to be adequately re-interpreted to accommodate the software testing knowledge and practices in the conformity assessment activities.

Due to the possibility that developers incur biased tests, the conformity of software is best evidenced when evaluated by an independent third party [32]. Several examples are successfully described in the literature, such as aviation systems conformity assessment using the DO-178C standard [33] and software evaluation based on IEC-61508 and IEC-61511 [34] standards for the functional and instrumental safety of a system. In the scope of information security, the Common Criteria (standard associated with ISO/IEC 15408 [35]) and FIPS 140-3 [24] are important examples of cases where the third-party evaluation applies to ensure the product quality and reliability.

In Brazil, for instance, there are the Conformity Assessment Programs (CAPs) regarding the digital time card (timesheet) machine (Portaria MTE/Inmetro 510/2015), the certification of products based on the Brazilian public key infrastructure, ICP-Brazil standards (such as cryptographic modules and smart card readers) and the regulation of software-controlled measurement instruments [25]. Particularly, software-based/embedded conformity assessment programs in Brazil have increasingly relied on independent evaluations by software analysis and testing accredited laboratories.

### 2.4. Software Analysis and Testing Accredited Laboratories

The term *software analysis and testing accredited laboratory* refers to a laboratory that demonstrated adequate levels of competence to be accredited in one or more software related scopes. These scopes are the combination among types of software-based/embedded products to be assessed (e.g., smart meters, smart card readers and temperature, pressure and humidity sensors) and the software verification or validation activities used to assess them (inspection, testing techniques, etc., also referred to as tests in traditional metrology). Each scope defines the required competences in performing a set of tests. For the sake of simplicity, terms such as *software analysis and testing accredited laboratory*, *software accredited laboratory*, *software code analysis accredited testing laboratory*, etc. are considered interchangeable throughout this manuscript.

### 2.5. Software Metrics and Techniques Applied to Interlaboratory Comparisons

In comparison to traditional areas of metrology, establishing metrics to compare the proficiency of laboratories involved in the assessment of software products is not a trivial task. As we show in Section 3.3, to the best of our knowledge, there is no formal method for conducting proficiency testing of laboratories accredited in scopes related to software conformity assessment.

In the United States, the NIST (National Institute of Standards and Technology) [27] manages the NVLAP (National Voluntary Laboratory Accreditation Program), which, through LAPs (Laboratory Accreditation Programs), establishes, develops and implements actions to accredit laboratories. Two LAPs relate to our theme, namely the Common Criteria Testing LAP and the Cryptographic and Security Testing LAP, but neither reports proficiency testing programs for accredited laboratories to date. In France, the ANSSI (Autorité Nationale en Matière de Sécurité et de défense des Systèmes d’Information) [28], which licenses external laboratories, validating their skills in technical security analysis for assessing the conformity of IT products, also does not conduct formal proficiency testing programs in order to monitor licensed laboratories. This scenario reinforces the relevance of the development of methods for prociciency testing for software analysis accredited labs.

The lack of solutions in the field of conformity assessment for “measuring” performance of labs’ evaluation team motivated the search for such metrics in SE. In the present study, two concepts were investigated with the goal of establishing metrics for proficiency testing: code coverage and software mutation.

#### 2.5.1. Code Coverage

Code coverage is a metric used in software test analysis to identify the number of lines of source code that have been tested [14]. The use of code coverage in our study is slightly distinct from how it has been seen in software engineering—when test cases and, in general, unit tests [36] are used to minimize errors and risks involved with the software development process [37].

Precisely, the proposed use of code coverage in our study is to present a pre-defined code coverage to a laboratory’s evaluation team and to challenge its members to find similar or equivalent test cases able to achieve that coverage. In other words, given a software source code and a set of lines covered by a reference unit test cases of the provider of proficiency testing round, the participant should try to cover the same set of lines, without knowing the reference and with a deadline for analyzing and delivering its results.

Thus, we considered code coverage is a promising concept for the comparison between laboratories and establishing a similarity measure between the effectiveness of the participant and the provider’s reference.

#### 2.5.2. Software Mutation

Software mutation is the introduction of minor changes to a program in order to investigate the effect in the behavior of software, generate variations that allow identifying the absence of inputs and, consequently, points of failure that the developed test cases could or should reveal. Variations in the original code are called mutants, and each mutant contains only one change from the original code. The modification is generated from a predefined set of mutation operators [15]. As the mutations involve specific changes in a single statement of software source code, in general, they imply a slightly different overall behavior of the software, which starts to present different responses for some sets of entries—naturally, those entries that will “excite” the modified statement.

We claim that the ability of a laboratory team to determine inputs that differentiate mutant software from the original is evidence of proficiency in code analysis, i.e., understanding part of software code to produce test cases able to excite the inserted defect.

In the present work, we use the so-called mutation score—which is the measure of the ratio between the total number of mutants killed (with the test cases developed by the laboratories) and the total number of mutants generated—as a metric for the comparison between laboratories.

#### 2.5.3. Theoretical Evaluation

Researchers proposing a new metric have the burden of proof to demonstrate that the metric is acceptable in its intended use. For almost half century, SE researchers have debated what would constitute a “valid” metric, i.e., a metric validated by a multifaceted, scientific and objective process. However, there must be a formal system of rules for ensuring such a process. SE community has not yet reached a consensus on this system of rules [38]. However, many studies have tried to ground their metric evaluations based on the Weyuker’s properties [39] (see, e.g., [40,41,42,43,44,45]).

Nevertheless, we could not directly use these properties to establish a point of view because they refer to complexity metrics, a product attribute. Such a fact could be a threat to internal validity of our study, since no evaluation would guarantee the metrics measure the attribute they purport to measure. In our case, the attribute we expect both metrics measure is the effectiveness of the participant in producing adequate test inputs with respect to specific goals or challenges.

In our preliminary investigations, we found Weyuker’s other work, which is well-known in the software testing technical literature [46], comprising a general axiomatic theory having 11 other properties in order to provide a set of adequacy criteria for evaluating software test measurements. The properties defined by Weyuker [46] are among those to which a “valid” measurement should adhere, according to Meneely et al. [38].

Fortunately, Weyuker [46] presented the theoretical evaluation related to both software test measurements in our study. Thus, we are not proposing to evaluate the previously mentioned metrics. Instead, we are providing a simple indication of an evaluation already performed by a specialist. According to the author, code coverage satisfies five and software mutation satisfies eight of properties defined by Weyuker [46] (11 in total). This indicates that software mutation measurements theoretically tend to be more successful than code coverage measurements regardless of the use, since they are directly or intuitively related to the attribute it aims to measure.

However, code coverage demands less effort than software mutation in terms of learning curve, instrumentation, etc. Thus, to evaluate code coverage measurement in the context of software conformity, an interlaboratory comparison would still make sense, at least as a way to evaluate and produce indicative evidence.

## 3. Research Method

### 3.1. Research Questions

In our first ad-hoc investigations, we were not able to identify well-reported and structured proficiency testing methods in the context of ICT or software products. It motivated us to propose initial open questions and, consequently, a research method for developing proficiency testing methods for software conformity assessment. Thus, with the purpose to minimally demonstrate our point of view, we summarize simple research questions, as follows:

**RQ1.**  *What is the available evidence about proficiency testing and interlaboratory comparisons in the technical literature in the last five years?*

**RQ1.a.**  *What part of the found evidence refers to ICT and software products’ conformity assessment and what part to sensor-based products’ conformity assessment?*

**RQ2.**  *Is it feasible to perform software product-related proficiency testing rounds by using quantitative performance measurement?*

### 3.2. Overview

**Figure 2:**
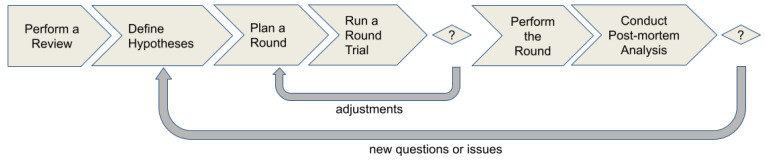
Research method.

The research method used in our study aims at: (1) acquiring and organizing an initial set of evidence on recent proficient testing methods; (2) if possible, identifying which evidence is related to sensor/ICT/software-based products; (3) capturing insights that could be reused or adapted in the context of software conformity assessment; and (4) organizing a set of steps that may permit performing some rounds of interlaboratory comparisons. Figure 2 illustrates the flow of the research method.

It is an iterative method and consists of six empirically performed steps, as follows:

1. Perform a Review: In this step, our goal is minimally to identify the most recent and available evidence regarding proficiency testing methods and which elements of them could contribute in developing a proficiency testing method in the context of software conformity assessment.
2. Define Hypotheses: Here, the purpose is to find or develop the theoretical and practical elements that could support defining some hypotheses to evaluate and determine which conditions make performing proficiency testing rounds in the context of software conformity assessment feasible.
3. Plan a Round: Based on the found elements and defined hypotheses in the previously performed step, a plan is designed and elaborated taking into account all requirements for performing an interlaboratory comparison or proficiency testing round according to the current version of ISO/IEC 17043.
4. Run a Round Trial: In this step, we expect to obtain the first indications of the round’s feasibility based on the planning. In general, students and collaborators not allocated in an accredited lab are recruited as participants in this step. If there are practical problems identified during the trial execution, they are analyzed and the adjustments are properly reported in the plan. Otherwise, the plan is considered ready and the round can be performed in the next step.
5. Perform the Round: The round’s plan is presented to the laboratories, and they are invited to act as participant of the round. Afterward, the round is performed and the participants’ data are collected.
6. Analyze Data and Publish Results: In this step, we analyze laboratories’ data, compute their performance and publish the results. In addition, we perform a post-mortem analysis aiming to synthesize lessons learned, identify new issues or questions and evaluate the theoretical and practical elements that could be removed or adapted and the new ones that must be investigated and incorporated. Thus, new hypotheses are stated and tested in the next iterations.

Currently, only two iterations (rounds) have been successfully performed at the writing of this manuscript. The main results of the two interactions of the research method (the review and the performed rounds) are presented in the following sections.

### 3.3. Literature Review

#### 3.3.1. Context

To support the existence of an open question on which we are basing this research work, we describe below the results of a *rapid review*. Similar to systematic reviews, a rapid review has its origins in medicine, it should be repeated and replicable and its results should be traceable. Instead, rapid reviews are a form of knowledge synthesis in which components of the systematic review process are simplified or omitted to produce information in a timely manner [47]. Tricco et al. [48] highlighted that there is no pre-established format for performing rapid reviews—even in medicine—and they have been also successfully exploited in computer science [49,50]. (In the future, we intend to plan and design a systematic review protocol to better understand and map the gap between research and practice regarding proficiency testing programme for ICT and software product conformity assessment; however, the results of such systematic review would be out of the scope of the present paper.)

#### 3.3.2. Search Strategy

Scopus database is the initial and unique information source to perform the search, because there is an intersection and, consequently, a lot of duplicate returned articles, when compared to other databases, such as IEEE Xplore, Engineering Village, ACM Digital Library, etc. [51].

Our search strategy is only to search in the title and abstract of papers written in English for the chosen search engine. In addition, the search string was formulated by combining well-known terms with the desired interval based on publication year of returned articles, as can be seen below:

TITLE-ABS-KEY ( (“proficiency testing” OR “interlaboratory comparison”) ) AND ( LIMIT-TO (PUBYEAR, 2021) OR LIMIT-TO (PUBYEAR, 2020) OR LIMIT-TO (PUBYEAR, 2019) OR LIMIT-TO (PUBYEAR, 2018) OR LIMIT-TO (PUBYEAR, 2017) OR LIMIT-TO (PUBYEAR, 2016) ) AND ( LIMIT-TO (LANGUAGE, “English”))

Note that we did not impose limits to returned papers on criteria based on terms discriminating any application area or domain, with the purpose of verifying whether there are ongoing or similar studies with respect to ICT or software conformity assessment reported incorrectly or inappropriately in an unknown or restricted forum. The work of Machado et al. [19] was used as the control study, i.e., it should also be found and included in subsequent review trials.

#### 3.3.3. Study Selection Procedure and Criteria

Our review’s selection procedure was divided into three phases: (1) performing the search in databases; (2) excluding articles based on the title and abstract; and (3) including articles based on the full reading. The criteria we used to judge the articles returned by the search engine and therefore decide on their pertinence and inclusion were as follows:

- Obtain the returned scientific publications searched in peer-reviewed journals and conferences available on the web through search engine, as evidence of proficiency testing in the last five years.
- Use Scopus’ pre-defined search filters to select for reading of title and abstract of the studies labeled as computer science or engineering, as the most probable subject areas to find ongoing or similar studies related to proficiency testing for sensor-, ICT- or software-based products’ conformity assessment.
- Select for full reading the papers that discuss ongoing or evaluated methods for performing proficiency testing for sensor-, ICT- or software-based products’ conformity assessment.

#### 3.3.4. Performing the Searches

The search was performed on 10 February 2021, and the total number of returned entries was 1310. Based on the selection procedure and criteria, all of these entries were selected. Table 1 shows that most of them refer to medicine, biochemistry, genetics, molecular biology and engineering (note that there are articles labeled in more than one area).

#### 3.3.5. Data Extraction and Review Results

According to our second selection criterion, the total number of articles selected for reading of title and abstract was 282. Surprisingly, computer science was able to return 52 papers. After reading the title and abstract of computer science-labeled articles, we found most of them refers to: (1) proficiency testing and interlaboratory comparison for electromagnetic compatibility measurements (14 articles); or (2) the use of algorithms and computer methods to support analyses of comparisons results (11 articles). When looking at engineering-labeled articles, we found 23 additional articles related to electromagnetic compatibility. Among these 282 articles, only six articles mentioned the word “sensor” in the title or abstract (four articles, a book and a conference proceedings’ report).

Unsurprisingly, only one was selected for full reading and included, in the protocol’s results, the control one—the first position paper regarding the subject of our research group in Brazil—preventing a more detailed analysis due to lack of data.

#### 3.3.6. Threats to Validity of Review and Preliminary Results

The main threats to the validity of this review are the researcher bias in selecting and including articles to the review’s results and the fact we limited the databases and period during performing the search. The research bias was minimized by strictly using the Scopus’s schema for the classification of articles in subject areas and by including for reading of title and abstract all returned articles. Furthermore, the search string did not include restrictive terms, such as software, system, information technology, sensors, etc., which makes the search broader. The limitations on choosing the database and time interval are notably a threat to the result’s generalization, and, consequently, we cannot argue that indeed there is no proficiency testing method-related ICT and software conformity assessment. However, this review shows even more traditional knowledge domains are currently investigating and developing new methods for proficiency testing and interlaboratory comparisons, aiming to address their open issues. Thus, we consider this review plays its role in this work. It provides some evidence and supports our belief that, if such methods exist, they are not adequately reported and easily available in the technical literature.

During the reading of title and abstracts phase, some works had our attention and deserved a full reading, because they point out or address issues or scenarios that could be appropriately matched to ICT or software proficiency testing methods by analogy.

For instance, Kotyczka-Moranska et al. [52] reported proficiency testing to determine gypsum oxide parameters in Poland. They highlighted there are difficulties in the selection of suitable indicators and criteria to evaluate the findings when the number of participants is small. They also claimed the performance evaluation criteria were determined from the participants’ results due to the impossibility of using a valid procedure according to more conventional procedures. Zavadil [53] showed that proficiency tests according to ISO 17043, for non-destructive tests laboratories, are effective to select competent laboratories and relevant to evaluate the continuous improvement environment of laboratories according to ISO 9001.

Furthermore, de Medeiros Albano and Ten Caten [54] analyzed the relationship among proficiency testing, validation methods and estimation of measurement uncertainty. They carried out qualitative research involving experts from five countries and concluded the tests contribute effectively to the reliability of the results and are directly related to validation methods and the uncertainty measurement in order to increase the laboratory’s competitiveness. In turn, Fant et al. [55] explored the proficiency testing of programming skills in computer science and electrical engineering courses. Although this work is related to computer science, it has a different goal, that is, to grade the students’ proficiency in their academic skills.

In [56], the researchers evaluated the capacities of pressure calibration laboratories by using a mobile measurement method. They emphasized that the results reported by testing laboratories were comparable, even though the measurements are often obtained using different methods.

According to Miller et al. [57], proficiency testing programs for patient care assessment are part of a rare category due to some restrictions: lack of reference measurement procedures, absence of certified reference materials, inability to prepare switchable samples, etc. In addition, Miller [58] discussed the problem of the dichotomy between using switchable samples and defined values in accordance with a reference measurement and using consensus approaches to standardize or harmonize the results of measurement procedures among the participants. Based on our briefly acquired experience, proficiency testing for ICT and software conformity program faces the same restrictions and problem.

Regarding the results of the research on sensors, in the book “Descriptive Analysis in Sensory Evaluation” [59], the authors stated that the performance checks on the performance of any sensory panel is critical. They highlighted the importance of proficiency testing in the evaluation process in several panels of sensory profiles, where the results of a common sample set and the output of each panel are compared with an expected output to validate performance, an action similar to the one we perform in our first round of interlaboratory comparison. The IEEE International Symposium on Electromagnetic Compatibility and Signal/Power Integrity 2020 conference proceedings [60] contains 137 articles and the topics discussed that most closely match our research include: experimental evaluation of spatial resolution for optical electric field sensors with dipole element and a schematic of proficiency testing of disturbance conducted at the mains terminals at 150 kHz and 30 MHz using multi-items.

In other articles found, Bair [61] commented that the traceability of gas flow in the range of 0.1–1 sccm (standard cubic centimeters per minute) is based on the extrapolation of the use of laminar flow elements below 1 sccm, and this part of the range has never been fully verified through interlaboratory comparisons, proficiency tests or other means of measurement assurance. Measurements needed to be made in absolute mode and the internal piston position sensor supplied with the piston gauge were not sufficiently accurate; thus, to support this range of gas flow within the scope of accreditation from Fluke Calibrations, a method needs to be developed to gain the necessary confidence for accreditation, a situation similar to what we try to do in our work for establishing a method for evaluating software products.

In [62], the authors reported their investigations of new sensors, new calibration facilities, investigation of the characteristics of the sensor, etc. This study developed and carried out interlaboratory comparisons during such investigations. As a consequence, the final report of the comparison was submitted to the World Meteorological Organization (WMO) and published as a report in the field of temperature, humidity and pressure on Instruments and Observation Methods Programme (WMO-IOM). According to the authors, the protocol will also be extended to other regions, and the results of all potential comparisons can be associated with the results in the European region, allowing the comparability of meteorological laboratories from different regions.

In [63], the authors proposed an interval data fusion procedure that can be widely applied in interlaboratory comparisons, prediction of fundamental constants, conformity tests, improving the accuracy of multi-sensor readings, etc. Their numerical experimental investigations show that using the proposed merger guarantees greater precision and robustness of the results of the interval data fusion procedure.

Finally, Sauerwald et al. [64] presented a gas sensor system for the detection of benzene, using Metal Oxide Semiconductor gas Sensors (MOS) and Temperature Cycled Operation (TCO). The system was equipped with three gas sensors, an advanced temperature control and electronic reading for the extraction of resources from the TCO signals. The system can be successfully calibrated in different laboratories and test conditions, indicating that the very different methods of generating benzene produce similar levels of test gas. The results demonstrate the need to define common test standards for trace gas sensor systems and the high potential of these systems for the quantitative detection of even small levels of pollutants such as benzene.

As we can see, there are some concepts, goals or tools that could be directly absorbed or adapted, and some similarities and analogies could be done to sensor- or software-based product conformity assessment. Nevertheless, new issues need to be identified and adequately addressed. In our opinion, the main issue is to determine how to quantitatively measure the accredited labs’ performance. It implies identifying or defining measures directly or indirectly related to the observed phenomena, and which can be easily comprehended and shared by different stakeholders (governments, regulators, technology producers and third-party evaluators).

## 4. First Round: Software Analysis Interlaboratory Comparison via Code Coverage

### 4.1. Rationale

As discussed above, one of the biggest challenges in establishing a model for comparing software testing laboratories is the difficulty in establishing quantitative measures due to the degree of subjectivity associated with the interpretation of the requirements of a software product. Thus, the first round of interlaboratory comparisons was based on code coverage measurement as the main element of objective comparison of the results obtained by each laboratory. Besides, each laboratory had the opportunity to evaluate its methods and procedures and verify the harmony with the performance of other laboratories [19].

Code coverage is a metric used to identify the quantity or set of “elements” of a software code that was tested by a given test case suite. These elements are, for example, lines of code, instructions, blocks of code, functions and procedures and can consider both source code and executable code [36,65]. Throughout this article, we consider the code coverage based on lines of software source code, which is a metric traditionally used to assess the test cases effectiveness [14].

Test cases contain a set of input values, pre-execution conditions, expected results and post-execution conditions developed for a specific objective and condition [36,65]. In the case of evaluating the quality of a software test case (or test suite), the classic use of code coverage is based on the premise that, the greater is the coverage of a test suite, the better is its quality—since this means that most of the software has been run (and therefore tested) [66]. In this round, however, the main competence to be assessed is not the ability to test a large portion of software, but the (much more abstract) ability to understand the software. This is because evaluating software code requires a certain level of understanding in order to identify non-conformities (defects) that often manifest in, for instance, malicious behaviors and undeclared functionalities. Non-conformities are unlikely to be identified through exhaustive or brute force techniques, but rather through a careful analysis of the characteristics and subtleties of each type of software. Thus, we adapted the code coverage metric and used it based on the following premise:

*Given a piece of software and a set of its lines of code, to develop a test case that covers exactly that set of lines demands understanding about the software in question.*

Indeed, since “understanding” is not a directly quantifiable concept, we do not intend to measure the “level of understanding”. On the other side, it seems reasonable to assume (on the contrapositive) that the lack of understanding of the software makes it impossible to determine a test case that returns a given coverage.

For the establishment of comparison metrics, however, we need to establish a formal hypothesis. Thus, we consider that not only developing test cases reaching a given coverage indicates a level of understanding regarding how to interpret a source code, but that:

**Hypothesis** **1.** *The closer a developed test case is to a given “target” code coverage, the higher is the understanding of its meaning and, consequently, how to test the software code.*

At this point, we define more precisely the concept of “software understanding” and, consequently, the meaning of the comparison based on code coverage.

We define the concept of “software understanding” as a partial order. This approach is consistent with the observation that it is not always possible to compare the “understandings” of two software analysts. In general, two people or laboratories may have incomparable understandings, for example, because they have specialized on different aspects of software. On the other hand, one subject having greater understanding than the other seems reasonable in certain circumstances, i.e., the possibility of determining that an entity may achieve “greater understanding” than the other is a premise for the laboratory comparison we propose in this round.

When defining a metric for comparison between two code coverages, two requirements must be met: (1) their similarity should increase when their intersection is larger, and (2) their similarity should decrease when their symmetric difference is larger. A simple metric with these properties is the Jaccard Index, defined by J(A,B)=A∩A/A∪B. The Jaccard Index can be rewritten as J(A,B)=A∩B/(A∩B+A∖B+B∖A), which makes it clear that the index is only 1 if A=B, being null if the intersection is null.

The Jaccard Index is a metric consistent with our definition of software code understanding in the following way. Consider a target coverage C in software with set S of lines and two analyst coverages $C_{1}$ and $C_{2}$. Suppose $\left(C_{1}\cap C\right)⊃\left(C_{2}\cap C\right)$, meaning that $C_{1}$ correctly covers all the lines of C that are covered by $C_{2}$ (and possibly more lines). In addition, suppose $\left(C_{1}\cap \left(S\setminus C\right)\right)\subset \left(C_{2}\cap \left(S\setminus C\right)\right)$, meaning that $C_{2}$ incorrectly covers all the lines of S∖C that are covered by $C_{1}$ (and possibly more lines). This is a clear scenario where $C_{1}$ evidences better software understanding than $C_{2}$—and the reader will be able to check that $J\left(C_{1}\right)\geq J\left(C_{2}\right)$, because of the assumptions regarding $C_{1}$ and $C_{2}$ and the definition of J(·).

One should note that the above metric is just one possible reference. Used in isolation, and with only a reference coverage, it is possible to inaccurately infer the difference among the levels of understanding of analyzers (laboratories). The scenario is analogous to a test that seeks to assess knowledge about a very wide field through a small number of questions. However, the idea is that, when testing a large amount of reference coverage, an increasingly accurate estimate of individual understanding levels is achieved and, consequently, more easily discriminated against.

### 4.2. Interlaboratory Comparison Execution

Based on the principle that code coverage provides a quantitative objective for interlaboratory comparison, we organized an interlaboratory comparison round attending all the requirements of ISO/IEC 17025:2017(E). Henceforth characterized as the Proficiency Testing Round, we describe in the following all aspects of the Proficiency Testing Round that needed to be defined prior to the execution of the round:

- Metric: As discussed above, we used code coverage to compare laboratories. We presented software together with a code coverage (arising from a real test case that was not informed to the labs) and challenged the labs to design test cases that achieve a code coverage as close as possible to the original one. To measure the distance between to coverages A and B, we used the Jaccard Index (A∩B)/(A∪B).
- Testing Item: The testing item was informed prior to the release of the code coverage challenges, so that the laboratories could have time to become familiar with the item. The chosen software was the available open source software Alliance Peer-to-Peer communication software Version 1.0.6 (build 1281) (http://alliancep2p.sourceforge.net/, accessed on 24 May 2021. ).
- Code Coverage Tool: The tool required to trace software execution and register code coverage were also informed prior to the release of the code coverage challenges. We used EclEmma JaCoCo 3.1.2 Plugin for Eclipse Java Platform [67].
- Delivery Mechanisms: A relevant—and new—property of the round is that, unlike “classic” proficiency testing that requires the physical transportation of a reference testing item/specimen, our round could benefit from Internet communication for transmission of the test item. Thus, we developed a virtual machine with the complete environment and tools needed to perform the software tests. To avoid problems due to the transmission of a large virtual machine, an encrypted packet was released one week before the beginning of the tests, so that, on the first day of the tests, the only thing needed was to release a decryption key on the website of the Proficiency Testing Round.
- Approval Criteria: To approve a lab in the challenge, its Jaccard Index of similarity should be larger than the mean Jaccard Index (among all participants) minus three times the standard deviation.
- Data Integrity: To assure the integrity and authenticity of data, all the communication between our organizing team and the laboratories were digitally signed with private keys corresponding to public keys that were securely exchanged before beginning the Proficiency Testing Round (in a registration stage). The chosen algorithms were SHA256+RSA2048.
- Cronogram: The Proficiency testing was a five-month process that started on 16 June 2019 with the elaboration of the work plan and finished in 20 December 2019 with the release of certificates of participation for the labs. The execution of the tests by the labs started at 10 a.m. (UTC-3) on 23 September 2019 and the deadline for return of the test reports by the labs was 4 p.m. (UTC-3) on 27 September 2019.

It is important to emphasize that all the above criteria were released to the labs on 27 August 2019, prior to the execution of the proficiency testing.

The resources needed to carry out the tests of this first round of EP were made available through a virtual scenario. Hence, the National Research and Education Network (RNP) (https://www.rnp.br/, accessed on 22 May 2021. ) was used, which is an environment for the safe transmission of data. Afterward, a digital test package was created according to the OpenPGP standard, using a private key (IPriv) generated specifically for the round.

The so-called Proficiency Test Package is a Virtual Machine (VM) that has the tools and files for the execution of the tests by the labs—all properly installed and configured (the package is available for download at https://doi.org/10.5281/zenodo.4781618, accessed on 24 May 2021). The virtual machine (a file with extension .ova) had to be run on a host with Oracle VirtualBox software version 6.0.8 installed. It was developed to facilitate participants’ access to these files and tools and the settings needed to run the tests. The package was prepared and delivered ready to use, together with operating instructions. The list below describes the content of the virtual machine and released to labs:

- JaCoCo 0.8.3. Java library for code coverage. More details about the library and how it was used in this research are presented below.
- Reference reports. These are the JaCoCo reports, in xml format, issued from the execution of the tests prepared by our organizing team.
- EP item. It consists of the source code and a functional version of Alliance P2P—Version 1.0.6 (build 1281).
- Auxiliary tools / files. The auxiliary tools or files available to facilitate analysis reports:
  1. Eclipse IDE 2019-03 (4.11.0). It is a platform with features and tools to streamline the software testing development process (it can be downloaded at: https://www.eclipse.org/downloads/packages/release/2019-03/r, accessed on 24 May 2021).
  2. EclEmma JaCoCo 3.1.2. Plugin based on the JaCoCo code coverage library for the Eclipse platform.
  3. Java-8-Openjdk-amd64. It is a development kit for Java platform systems, the software programming language used in this round.
  4. Junit 5. It is a framework used to facilitate the creation of unit tests in Java.
  5. comparaRelatorios.py. It is a script developed in Python programming language, which automatically compares the lines covered, not covered and covered incorrectly, in the report of the appraised with the reference report. It was made available to the participants of the round to facilitate the identification of divergent and convergent lines.
  6. zeresima.xml. It is a JaCoCo report, in xml format, formed by zeroed lines, simulating a unit test that does not cover any line. When used with the script comparaRelatorios.py, zerezima.xml is compared to a reference report, pointing out the diverging lines, that is, the lines that are not zeroed and that, obviously, were executed by the reference test case. Note that the VM for the Proficiency Testing Round can be downloaded from the linked Virtual Machine and all the additional details of the round can be obtained in Proficiency Testing Round (in Portuguese).

#### 4.2.1. Code Coverage Library—JaCoCo

**Figure 3:**
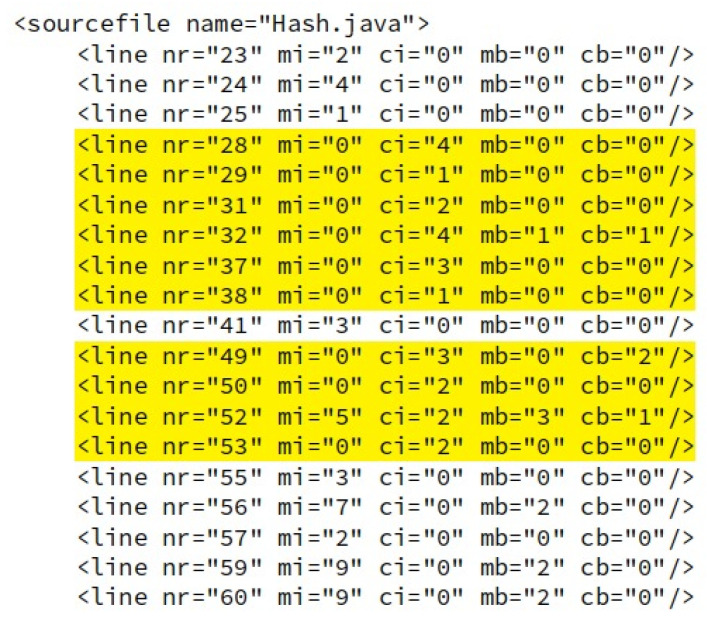
Sample from JaCoCo report.

A Java tool, JaCoCo records the coverage of a test in reports, which can be issued in HTML, XML and CSV format files. The report in xml format indicates the number of lines and branches covered and non-covered of each test case in textual format and is the extension adopted in the Proficiency Testing Round. Figure 3 exemplifies an excerpt from the xml report of the test coverage of the Hash.java class. In this image, it is possible to analyze the section of lines 23–60, where the instructions covered by the HashTest.java test case are highlighted in yellow.

In the XML report, the <line> tags, within the <source file> tags, present information about the instructions and branches covered and not covered for each line of code. Branches are structures of code conditions, where the previous action determines which decision the software will execute. A branch is lost when only part of the condition is satisfied. The attributes of the <line> tag are: line number (nr), missing instructions (mi), covered instructions (ci), missed branches (mb) and covered branches (cb).

#### 4.2.2. Unit Tests—JUnit

**Figure 4:**
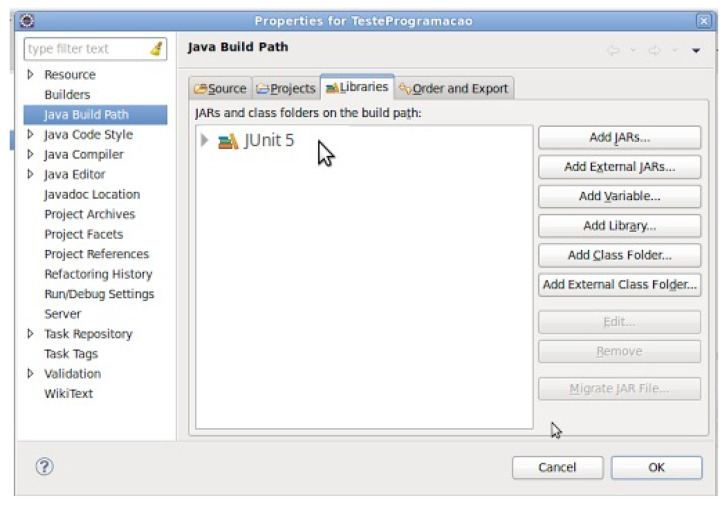
Adding JUnit to Eclipse.

The choice of using the Jacoco library in this round led us to use the JUnit framework in the creation and execution of unit tests. In this round, the JUnit 5 version was used and supported by Eclipse IDE 2019-03. Thus, it was enough to configure the framework in the Eclipse project, as can be seen in Figure 4.

During the planning and design, eight test cases were elaborated, of which seven were used for the evaluation of the round. The test case not used, named HashTest.java, was sent to the laboratories within the digital package and described in the proficiency testing manual. Its purpose is to exemplify how the participants should perform the test of this scheme. Each test case was concerned with testing a class of the Java source code for the item. Thus, the criteria for choosing these classes started from the following premises:

- The classes should belong to different packages of the Core subsystem of the item, avoiding the test cases needed cover graphical user interface (GUI) functionalities.
- The classes should allow tests of different levels of difficulty.
- The classes should test decision structures and cover false and true conditions.

#### 4.2.3. Software Alliance P2P

The software adopted as an item for the first round was Alliance P2P, an open source technology (open source) designed to share files and establish communication between close people. In JAVA programming language, Alliance is composed of two independent subsystems: (1) UI, responsible for the GUI; and (2) Core, responsible for the internal part of the system. The Core subsystem is divided into three packages:

- Comm contains the classes responsible for the ow of network data.
- Node contains classes with information from actors (users who share files).
- File contains classes for managing shared files.

**Figure 5:**
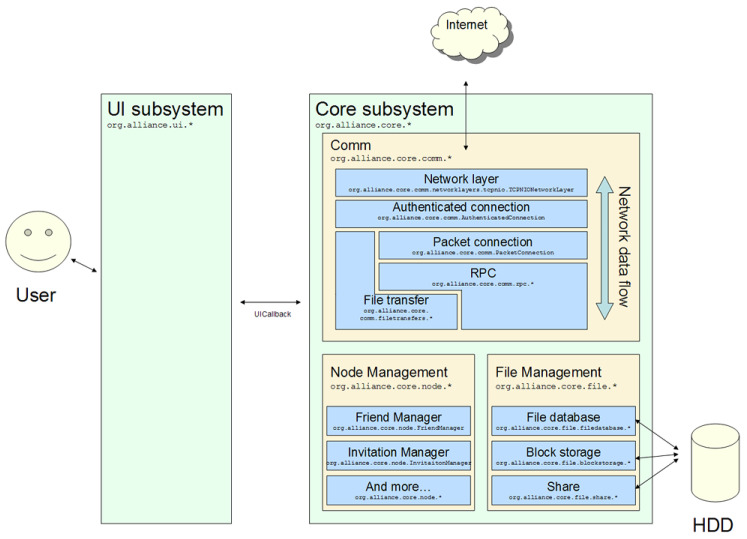
Alliance P2P architecture overview.

Each of these packages should be linked to at least one test case. Figure 5 illustrates an overview of the Alliance P2P architecture.

### 4.3. Evaluation Criteria

Since the objective in this round was to find test cases that covered exactly the same lines of code covered by the reference tests, we needed to propose an evaluation mechanism. Thus, the participant’s performance would be better when its test cases’ results were more similar to the reference tests. Then, for a given test case based on code coverage, *T* is called the total set of lines of the evaluated software, $S_{R}$ is the set of lines covered by the reference tests and $S_{L}$ is the set of lines covered by the tests performed by the participant. As a similarity metric used to measure the performance of each participant, the Jaccard Index [68] was adopted, defined below:

(1) $$J\left(S_{R},S_{L}\right)=\frac{S_{R}\cap S_{L}}{S_{R}\cup S_{L}}$$

The determination of the consensus value of this interlaboratory comparison was based on the average of the $J(S_{R},S_{L})$ indices obtained by each of the participants. Thus, the value $\underline{J}$ is given by:

(2) $$\underline{J}=\Sigma_{i=1}^{labs}\frac{J(S_{R},S_{L}^{i})}{labs}$$

where *labs* is the number of participants. The interpretation of the performance of the *i*th laboratory in relation to the other participants was associated with the comparison of its Jaccard Index, with the standard deviation, given by:

(3) $$\sigma =\sqrt{\Sigma_{i=1}^{labs}\frac{{\left(\underline{J}-J\left(S_{R},S_{L}^{i}\right)\right)}^{2}}{labs}}$$

The result of the evaluation of the index of the *i*th participant (Ji) was given by the following criteria:

- $J_{i}\geq \underline{J}-\sigma$ indicates “satisfactory” performance and does not generate a signal.
- $\underline{J}-\sigma >J_{i}\geq \underline{J}-3\sigma$ indicates “questionable” performance and generates a warning signal.
- $J_{i}<\underline{J}-3\sigma$ indicates “unsatisfactory” performance and generates an action signal.

The results of the Ji indices were rounded to two decimal places, following rounding criteria.

### 4.4. Participant Performance and Round’s Results

The Proficiency Testing Round included the participation of five accredited laboratories and one academic laboratory, all of which reported the adequacy of the proposed procedures: all labs could easily download the test item, perform their tests and return the corresponding reports.

Table 2 shows the results of each participant [69], measured by the Jaccard Index. As can be seen, all participants obtained maximum value, with index Ji=1. Such result implies that all participants were able to solve the challenge related to this round of the proficiency testing, and consequently implemented test cases that cover exactly the set of lines of the provider’s reference.

As a consequence of each laboratory having achieved the Jaccard Index of 1, the standard deviation was zero. Therefore, the application of the established criteria indicates that all participants had a satisfactory result.

Even though the the above result prevented a further comparison among the participant laboratories, such result was in a way foreseen. On the one hand, as we restricted participation to “high profile laboratories” in the Proficiency Testing Round (accredited labs and academic labs), the expected results were indeed high Jaccard Indices. It demonstrates that all participants have the minimum competence expected to perform tasks essential in software conformity assessment. On the other hand, since the results of this first Proficiency Testing Round would impact the accreditation of labs, we opted to develop relatively easy challenges. Given we focused on the feasibility of the round, the round’s result indicates that the next ones should propose more challenging test scenarios, in order to better explore the participants’ technical competence.

**Figure 6:**
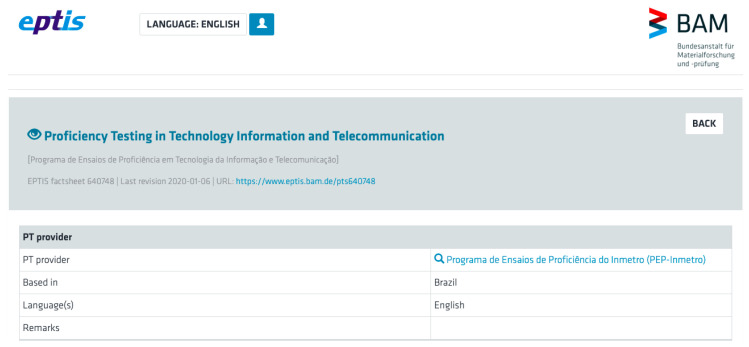
Registration of the proficiency testing in the EPTIS database.

As a result of following all the requirements of ISO/IEC 17025:2017(E), the Proficiency Testing Round was registered in the EPTIS database, as shown in Figure 6.

The full details of the registration of the Proficiency Testing Round can be obtained on the corresponding EPTIS webpage (https://www.eptis.bam.de/eptis/WebSearch/view/640748, accessed on 22 May 2021).

## 5. Second Round: Interlaboratory Comparison Using Software Mutation Metrics

### 5.1. Rationale

Despite the encouraging results of the Proficiency Testing Round carried out in 2019 with respect to the feasibility of the proposed method, the fact that all laboratories obtained the same Jaccard Index 1 prevented the realization of a detailed statistical comparison among them. As discussed above, we avoided higher difficulty challenges to reduce the risk of laboratories failing. After the conclusion of the first Proficiency Testing Round, we decided to plan a new round, exploring different tools and methods.

Following the strategy of looking at SE discipline, we investigated whether the concept of software mutation could be explored in order to use other metrics. Recall that, given the code of software *S*, a mutant $S^{\prime }$ of *S* is slightly distinct software where just a statement of code or instructions are modified, and an input *I* kills the mutant $S^{\prime }$ if *S* and $S^{\prime }$ present distinct behavior when presented to input *I*.

In addition, it is interesting to mention that the mutation testing technique is closely related to the code coverage technique. Note that, given S software and $S^{\prime }$ mutant, for a given test to be able to “kill” the $S^{\prime }$ mutant, this test must necessarily cover at least the statement that distinguishes *S* from $S^{\prime }$. However, it is not enough that such a statement is covered: the change could generate different behaviors between the original line and the modified statement. Thus, in some way, mutation testing presents a possibly even stronger method to assess software analysis and test effectiveness.

Thus, we define the mutation score $s_{i}\in \left[0.1\right]$ of laboratory *i* by the following equation:

(4) $$s_{i}=\frac{M}{T}$$

where *M* is the number of killed mutants and *T* is the total number of generated mutants. (Note that the traditionally employed approach would be to subtract from *T* the number of “equivalent” mutants, i.e., the generated mutants that present the same behavior as the original software. However, detecting such mutants is hard, and, for the purposes of the present research, it is more important to have a reference for comparing laboratories.) It makes the hypothesis of this round simpler than the one in the first round:

**Hypothesis** **2.** *When a higher the mutation score is obtained by a set of testing cases, its effectiveness of testing and detecting defects is better and, consequently, the understanding of the software is better as well.*

In other words, Hypothesis 2 states that labs’ performance can be discriminated by using a mutation score based measure.

### 5.2. Interlaboratory Comparison Execution

A new round of interlaboratory comparison was planned for the second half of 2020. This new round would have considerably greater complexity and a variety of challenges. To have flexibility in defining challenges, technologies and procedures, the organization committee of the round decided to perform an interlaboratory comparison instead of a proficiency testing—i.e., an interlaboratory comparison that does not follow all requirements of ISO/IEC 17043:2017(E). Since the round would contain challenges with complexity and unknown difficulty, it was essential to define or extend the data analysis procedures or approval criteria of participants a posteriori (recall that one of the requirements of ISO/IEC 17043:2017(E) is that all approval criteria must be defined before the proficiency test round is carried out, which is not convenient when searching for new (and unknown) comparison methods).

The choice of not meeting ISO/IEC 17043:2017(E) also allowed simplified procedures to be adopted in relation to the registration of laboratories and the processing of data, once there was no need to segregate teams in charge of receiving data from registrants and processing test results. A more particular issue in this round was the possibility of experimenting on external and individual participation (software developers, students, testing teams, etc.). This issue could increase the mass of data to be analyzed, but it would hinder performing an official proficiency test round.

In this round, the challenge proposed to the participants is that they produce test cases that efficiently eliminate the greatest possible number of mutations for the classes selected by the round’s organization committee, within the established deadline. The classes were selected considering the number of mutants generated by the PIT tool (the PIT tool (https://pitest.org/, accessed on 24 May 2021) was used through a plugin for the Eclipse IDE, which allowed a quick analysis of the quantities of mutants generated and killed by the test cases developed by the participants, through a summary generated by the plugin itself), adopting the complete set of mutation operators provided by it.

As done in the first round, the content of the package was made available on a virtual machine at a specific link with the fundamental and auxiliary tools and files installed and configured to be used. Many of the tools and files from the previous 2019 round could be used in this new 2020 round. However, we used specific tools to aid in the analysis of the mutation score and the participant’s performance. Here are lists of the fundamental and auxiliary tools and files.

- Fundamental tools and files:
  - Proficiency test item: source code and a functional version of the software product chosen for the round.
    * Alliance P2P—version 1.2.0 (build 1281).
- Auxiliary tool and files:
  - Eclipse IDE for Enterprise Java Developers—version: 2019-03 (4.11.0).
    * Java-8-Openjdk-amd64;
    * JUnit5; and
    * PIT (Pitest)—tool for mutation testing and code coverage.

The virtual machine was made available through a file with the extension .ova, and, to run it, the physical machine required that Oracle Virtualbox software version 6.0.8 was installed with a minimum of 10 GB storage space and a Core i5 processor with 6 GB of RAM. The operating system of the virtual machine was Lubuntu 19.04.

#### Mutation Testing—PIT (Pitest)

In this second round, some tools and the test item in the first one were kept, including Eclipse, JUnit and the Alliance P2P. To investigate whether, by changing the measure and challenges, there could be better discrimination between the participant’s performance, the round’s organization committee focused efforts on defining meaningful metrics and building interesting “challenges”. The more important change in the testing environment was precisely the software mutation tool.

**Figure 7:**
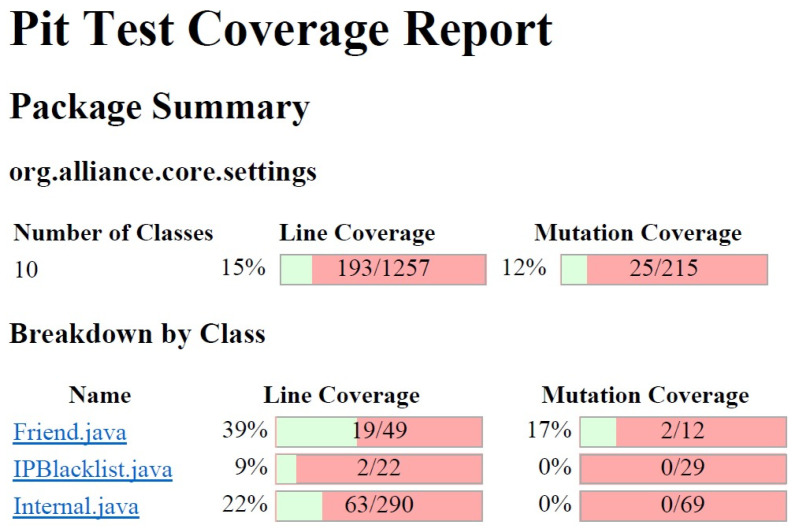
Example of a PIT report.

PIT is a mutation tool for JAVA language. It generates a large number of mutants based on a set of operators can be tuned. Besides, PIT presents statistics about the number of mutants that were killed by the test suite, as shown in Figure 7.

The performance of each participant is based on the relationship between the number of mutants killed and the size, in KBytes, of the set of tests developed. In this way, participants who were able to eliminate a large number of mutants with a reduced number of test cases would be more efficient. Based on this premise, it would be possible to conclude which participants would have a good understanding of the software documentation, the code related to those tests, how the tests should be performed and, consequently, conclude which participants would be proficient in the execution of code analysis and tests.

### 5.3. Evaluation Criteria

The determination of the consensus value of this round of comparison was through the average (μ) of the ratios between the mutation score and the size of the test cases of each participant, given by:

(5) $$\mu =\Sigma_{i=1}^{n}\frac{\left(s_{i}/t_{i}\right)}{n}$$

where $s_{i}$ is the mutation score and $t_{i}$ is the size of the test cases of the participant *i*, measured in Kbytes of the object code (Java bytecode), and *n* is the total number of participants.

The interpretation of the performance of the *i*th participant in relation to the others is associated with the comparison of his performance index with the standard deviation σ given by:

(6) $$\sigma =\sqrt{\Sigma_{i=1}^{n}\frac{{\left(R_{i}-\mu \right)}^{2}}{n-1}}$$

where $R_{i}=s_{i}/t_{i}$ is the individual performance indicator for participant *i*. The result of the evaluation of the index of the *i*th participant is given by the following criteria:

- $R_{i}>\mu +3\sigma$ indicates “exceptional” performance and does not generate a signal.
- $\mu +3\sigma \geq R_{i}\geq \mu +2\sigma$ indicates “very good” performance and does not generate a signal.
- $\mu +2\sigma \geq R_{i}\geq \mu +\sigma$ indicates “good” performance and does not generate a signal.
- $\mu +\sigma \geq R_{i}\geq \mu -\sigma$ indicates “satisfactory” performance and does not generate a signal.
- $\mu -3\sigma \leq R_{i}\leq \mu -\sigma$ indicates “acceptable” performance and generates a warning signal.
- $R_{i}\leq \mu -3\sigma$ indicates “unsatisfactory” performance and generates an action signal.

The results of the indices, presented in the following section, have been rounded to five decimal places, following rounding criteria, due to the fact that the $R_{i}$ values are very low.

### 5.4. Participants Performance and Round’s Results

This Proficiency Testing Round included the participation of laboratories, professionals, students and software organizations. For the calculation of mutation scores for each participant, the 2938 mutants generated by the PIT tool for the org.alliance.core.com.rpc package were considered. Table 3 shows the results of each participant. As the round held a public track, a group of students participated and submitted their solutions. Obviously, the collected data from this group were not considered for calculating the mean and standard deviation in conjunction with accredited laboratories.

With the data shown in Table 3, the mean (μ) and standard deviation (σ) associated with the values of the performance indices ($R_{i}$) were, respectively, 0.03185 and 0.05480. Therefore, the application of the established criteria indicates that all participants presented satisfactory results, except for the participant identified by “ID-4”, whose performance was classified as “good” [70].

## 6. Conclusions

### 6.1. Discussion and Final Considerations

Regarding the first two research questions (RQ1 and RQ1.a), this work performs a rapid review and brings up the lack of evidence regarding well-reported and evaluated proficiency testing or interlaboratory comparison methods in the context of intelligent sensors or software conformity assessment. This makes the comparison with other similar studies impracticable and points out there is an open question about how to obtain such methods through scientific knowledge production.

Analyzing and testing currently produced sensors requires exploring more complex scenarios, which primarily and naturally involves an adequate understanding of software issues (functional and non-functional requirements, integration capabilities, etc.) Hence, our expectation is to continuously use and evolve the presented research method for empirically investigating and developing both software- and sensor-related proficiency testing.

With respect to RQ2, the main contribution of this work is to demonstrate the feasibility of quantitatively measuring interlaboratory comparisons involving software analysis and testing accredited laboratories. Despite the evident importance of these comparisons, this is the first work that proposes a systematic approach to quantitatively measure, evaluate and evolve such activities in software conformity assessment. Two rounds of interlaboratory comparisons were carried out: a “more conservative” first round and a “more daring” second one.

An eventual failure on the part of the organization or an insufficient result on the part of laboratories could create obstacles for the accreditation process and for the very support of the nascent network of accredited laboratories in software analysis. Thus, we chose to follow a “conservative” approach in the first round and conducted a round meeting all the requirements of a proficiency test as provided for in ISO 17043, restricted to accredited and academic laboratories. On the other hand, we chose to present as a “challenge to the laboratories” a set of test cases having only moderate difficulty, reducing the risks that there would be bad results on the part of the laboratories. In addition, there was no clear perception of how the laboratories would behave in such a round, and whether the proposed challenges really reflected activities associated with the competencies of those laboratories. The completion of the round represented a great challenge, because carrying out the round was a totally new process. The successful execution of the round produced lessons learned and gave us confidence in the procedures, from the comparison methods and metrics to the procedures of disclosure, delivering instrumentation and test items and receiving/processing of results. The result of this first round is described in Section 4.4: all laboratories achieved a Jaccard Index score of J = 1. This prevents the labs’ performance from being used to discriminate them through a more in-depth comparative analysis and cannot support Hypothesis 1. However, it is one of the first pieces of evidence of the feasibility of quantitative interlaboratory comparison related to software conformity assessment.

The results achieved in the first round allowed having confidence in the labs’ technical capacity. Thus, the second round exploits more freely other methods and metrics. In this context, we opted for the use of a mutation score-based metric, which could generate more test case scenarios. Due to this option, the second round did not define the data analysis and approval criteria a priori. As a consequence, the interlaboratory comparison round cannot be characterized as a proficiency test. On the other hand, being characterized as a rigorous comparison between the participating laboratories, the round continued to be a valid mechanism to meet performance monitoring requirement as a “different interlaboratory comparison of proficiency testing", under the terms of item b of article 7.7.2 of ISO/IEC 17025:2017. By dismissing meeting the requirements of ISO 17043, other aspects of the round could be simplified, such as the procedures for guaranteeing integrity and irrefutability and the mechanisms for identifying collusion. Additionally, such simplifications permitted holding a parallel and “public” track, admitting the participation of software organizations not actively involved in conformity assessment, practitioners and even students. It demonstrates the possibility of exploiting other experiment designs, recruiting new kinds of participants (by expertise, organizational arrangement, etc.) and new ways of data gathering. In addition, we believe the large number of classes to be evaluated and a short time for producing test cases were also important factors in ensuring that there would be variability among the participants’ performance. Finally, Hypothesis 2 is supported by the second round’s result and is the second and more concrete piece of evidence of the feasibility of quantitative interlaboratory comparisons related to software conformity assessment provided by this work.

In contrast to the first round, the test cases and reports delivered by labs indicated the proposed challenge in the second round was more difficult. According to some returned reports, this difficulty would be related to the need to deepen both the inspection of the source code and the design of test cases. In addition, the test item to be developed in the Java language was reported by one of the participants as being an activity outside the scope of services provided by laboratories. However, the setbacks brought by a programming language are problems inherent to the labs performing software conformity assessment. In general, many real software products are composed of different technologies according to the manufacturer’s choice and are not limited to the technologies dominated by accredited laboratories. In addition, the use of mutation testing was indicated to be more effective, thus it required greater dedication from the participants in the steps of code inspection and testing. This fact could probably be stated as a new hypothesis (“developing test cases to kill mutants tends to be more complex than that ones just covering the same set of lines of software code”). In the future, we are going to evaluate whether it would deserve more attention and whether its investigation is into the scope of our research.

### 6.2. Future Works

The present work describes a first approach to interlaboratory comparison for evaluating software analysis and testing accredited laboratories. Hence, we foresee a series of possible outspreads of the present project, as we describe in the following.

- Specific purpose software: In the executed rounds, a generic network application was used as the test item. The idea is that the analysis of such software would not involve too specific knowledge in any application area, being, therefore, more suitable for initial rounds of interlaboratory comparison. In future rounds, one can explore the analysis of software modules dedicated to specific applications within the scope of the participating laboratories. For example, accredited laboratories for testing software for smart energy meters could be challenged to perform analysis of electrical metrology software modules.
- Security testing: In several conformity assessment programs, it is important that the test lab masters cybersecurity analysis techniques. One way to assess the competence of laboratories in cybersecurity is to carry out interlaboratory comparisons with reference to test cases that explore exploitation of vulnerabilities and security flaws.
- Integration tests: All the challenges presented to the laboratories in the two rounds explored highly compartmentalized test scenarios, such as unit tests (class tests). An interesting question is whether it would be possible to develop a comparison model between laboratories based on system or integration tests, that is, tests in which the software application is executed as a whole or closer to its totality. This scenario can bring a new level of complexity and difficulty that allows a more rigorous assessment of the competence of the participating laboratories.
- Standard model: There is currently no standard model for measuring laboratories performance in evaluating software products. Analyzing other technical areas, such as chemistry, biomedical, medicine, physical, etc. would be interesting to check what is worth bringing to computer science and what can applied in software companies, software development, software testing, etc.

### 6.3. Implications

#### 6.3.1. For Practitioners

In a national quality infrastructure, technical excellence of accredited labs is essential to guarantee that goods meet the security, safety and quality requirements. This means guaranteeing that such goods can be consumed by citizens, industries and nations, as well as that technology producers compete in fair and healthy economies. Even with the increase of demand in the last years, sensors, ICT or software conformity assessment has not accompanied the pace of knowledge production in other conformity assessment areas, taking into account the produced knowledge about how to measure this excellence in an impartial, objective and fair way.

This work is part of a project that started the collaboration among the national metrology institute and two universities in Brazil, aiming at investigating, developing and evolving processes, methods and tools to make that measurement feasible. As a concrete result for Brazilian society, the results of both reported rounds have been used to evaluate and harmonize the competence of the body of Brazilian accredited labs’, which are directly involved in evaluating real software-embedded products related to conformity assessment programs. Among these products, there are digital time card (timesheet) machines, smart card readers, smart meters, etc.

Additionally, one of that project’s goals is to organize and perform at least one round of interlaboratory comparison per year, which leads the project team to expand collaboration with other national or international institutions for: (1) building and spreading knowledge; (2) speeding up the development of methods, tools and human resources; (3) facilitating international comparisons and trades; and (4) empowering metrology institutes or nations.

#### 6.3.2. For Researchers

To respond to the first two research questions, we exposed the lack of evidence and the emergent need of producing high quality scientific content regarding sensor- and software-related proficiency testing and interlaboratory comparison methods.

To respond to the last research question, we proposed a research method and carried out two rounds of interlaboratory comparisons, which became in this article the first piece of evidence of the feasibility of quantitatively measuring performance of software analysis and testing accredited labs properly reported in the technical literature. Furthermore, all of our results lead us to believe that the concepts, metrics, procedures and result are replicable or repeatable, whether taking into account the necessary adjustments inherent to the particularities of each region, country or international consortium.

Thus, we conclude this paper claiming researchers and practitioners must consider establishing a research agenda for proficiency testing methods to support conformity assessment of sensors, ICT and software products. The lack of new research is a risk of any scientific discipline. It jeopardizes the building of news ideas and strengthens the prevailing ones, which prevents accelerating the pace of knowledge acquisition in a theme [51].

## Figures and Tables

**Table 1:** Returned articles grouped by subject area according to Scopus.

Subject Area	Number of Studies (Non-Exclusive)
Medicine	481
Biochemistry, Genetics and Molecular Biology	282
Engineering	264
Chemistry	260
Physics and Astronomy	225
Environmental Science	163
Agricultural and Biological Sciences	135
Chemical Engineering	109
Earth and Planetary Sciences	96
Immunology and Microbiology	92
Health Professions	82
Pharmacology, Toxicology and Pharmaceutics	60
Social Sciences	59
Materials Science	55
Computer Science	51
Energy	36
Mathematics	28
Multidisciplinary	19

**Table 2:** Results of the first round.

Participant Code	Jaccard Index
01	1.00
05	1.00
07	1.00
12	1.00
14	1.00
18	1.00

**Table 3:** Results of participants in the second round.

ID	Mutation Score	Killed Mutants	Bytecode (KB)	Ri
ID-2	2.11%	62	49.60	0.00043
ID-3	0.00%	0	9.60	0.00000
ID-4	63.36%	1832	6.51	0.09513

## Data Availability

The data presented in this study are available on request from the corresponding author.
